# Regulations as to the Storage of Poisons

**Published:** 1910-01-15

**Authors:** 


### Regulations as to the Storage of Poisons.

While definite rules relating to the storage of poisons are laid down, and must be complied with by all pharmacists, these regulations are not by law applicable to medical practitioners who dispense medicines. The rider which the jury at the Fulham Coroner's Court on January 4 added to their verdict in the case in which death was due to an unfortunate error in dispensing is, therefore, worthy of serious consideration. In the case in point the practitioner frankly admitted that the bottle in which the chloroform water was kept in his dispensary was unfortunately similar in appearance to the bottle containting solution of strychnine, and there can be little doubt that it was owing to this similarity that he made the mistake of using strychnine solution when he intended to use chloroform water. The jury expressed a desire that the Coroner would make a strong recommendation to the proper authorities, calling attention to the case and "the need for legislation to make it compulsory for poisons to be kept in bottles which could be easily distinguished from bottles containing non-poisonous substances." The regulations with which pharmacists are compelled to comply provide that in the keeping of poisons each receptacle containing a poison shall be labelled with some distinctive mark indicating that it contains poison; also that each poison be kept on one or other of the following systems: (*a*) in a bottle or vessel secured in a manner different from that in which bottles or vessels containing ordinary articles are secured; or (*b*) in a bottle or vessel rendered distinguishable by touch from bottles or vessels in which ordinary articles are kept; or (*c*) in a bottle, vessel, box, or package kept in a room or cupboard set apart for dangerous articles. The penalty for non-compliance with these regulations is £5. While, as already stated, medical practitioners are exempt from these provisions it seems desirable that they should comply with them voluntarily, and doubtless the vast majority of practitioners who dispense do so comply with them. Very great precautions are taken in the storage of poisons in institutional wards, and it is highly desirable that similar precautions should be taken in private surgeries. The regulations further provide that in the dispensing and selling of poisons, all liniments, embrocations, lotions, and liquid disinfectants containing poison shall be sent out in bottles rendered distinguishable by touch from ordinary medicine bottles. Compliance with this regulation undoubtedly makes for safety, and it would seem wise for practitioners to observe it, although they are not required by law to do so.

